# Genetic Analysis Workshop 15: gene expression analysis and approaches to detecting multiple functional loci

**DOI:** 10.1186/1753-6561-1-s1-s1

**Published:** 2007-12-18

**Authors:** Heather J Cordell, Mariza de Andrade, Marie-Claude Babron, Christopher W Bartlett, Joseph Beyene, Heike Bickeböller, Robert Culverhouse, L Adrienne Cupples, E Warwick Daw, Josée Dupuis, Catherine T Falk, Saurabh Ghosh, Katrina A Goddard, Ellen L Goode, Elizabeth R Hauser, Lisa J Martin, Maria Martinez, Kari E North, Nancy L Saccone, Silke Schmidt, William Tapper, Duncan Thomas, David Tritchler, Veronica J Vieland, Ellen M Wijsman, Marsha A Wilcox, John S Witte, Qiong Yang, Andreas Ziegler, Laura Almasy, Jean W MacCluer

**Affiliations:** 1 Institute of Human Genetics, Newcastle University, Newcastle upon Tyne NE1 3BZ, UK; 2 Mayo Clinic, Rochester, Minnesota 55905, USA; 3 INSERM U535, BP 1000, Villejuif, 94817, France; 4 Columbus Children's Research Institute, Columbus, Ohio 43205, USA; 5 Hospital for Sick Children, Toronto, ON M5G 1X8 Canada; 6 Universität Göttingen, 37073, Germany; 7 Washington University, St Louis, Missouri 63110, USA; 8 Boston University School of Public Health, Boston, Massachusetts, USA; 9 Washington University School of Medicine, Division of Statistical Genomics, St. Louis, Missouri 63108, USA; 10 Teaneck, New Jersey 07666, USA; 11 Indian Statistical Institute, Kolkata 700 108, India; 12 Case Western Reserve University, Cleveland, Ohio 44106-7281, USA; 13 Duke University, Durham, North Carolina 27710, USA; 14 Cincinnati Children's Hospital, Cincinnati, Ohio 45229, USA; 15 INSERM U563, Toulouse, 31024, France; 16 University of North Carolina, Chapel Hill, North Carolina 27514, USA; 17 University of Southampton, Hampshire SO166YD, UK; 18 University of Southern California, Los Angeles, California 94720, USA; 19 Ontario Cancer Institute, Toronto, Ontario, M5G 2M9 Canada; 20 University of Washington, Seattle, Washington 98195-7720, USA; 21 i3 Drug Safety, Waltham, Massachusetts 02451, USA; 22 University of California, San Francisco, California 94143-0794, USA; 23 Institute of Medical Biometry and Statistics, Lübeck, 23538, Germany; 24 Southwest Foundation for Biomedical Research, San Antonio, Texas 78245, USA

## Preface

This issue of *BMC Proceedings *contains the proceedings of Genetic Analysis Workshop (GAW) 15, which was held November 11–15, 2006, in St. Pete Beach, Florida, USA. The GAWs began in 1982 and are now held in even-numbered years. They provide a forum for investigators interested in identifying genetic effects on complex diseases to evaluate and compare novel and existing statistical methods. The purpose of these Workshops is to allow the comparison of statistical methodologies for genetic epidemiology using common, well described data sets. Prior to each GAW, topics are chosen, one or more existing data sets are selected, and a set of simulated data is created that permits investigation of current questions of broad interest in statistical genetics. These data are made available to any scientist who requests them, and their analyses of these data are presented at the Workshop. Participation in the Workshop is open to anyone who submits an analysis of one of these data sets, provides data, or participates in Workshop organization. More information about GAW, including details of upcoming Workshops, may be found at .

Three data sets (two empirical and one simulated) were distributed for GAW15, addressing two general classes of problems: 1) the genetics of expression, and 2) methods for dissection of complex traits. For the first time, one of the real data sets included RNA expression data from microarrays (Problem 1). Analyses of these data by GAW15 participants were focused primarily on expression data as quantitative traits in linkage and association scans and on methods for extracting additional information from the massively multivariate nature of the data set, which included literally thousands of quantitative traits. The second real data set (Problem 2) provided opportunities to address such methodological problems as separating multiple functional loci within a region of linkage or association, and linkage and association analyses of markers in the pseudoautosomal region of the X chromosome. The simulated data set (Problem 3) for GAW15 was based on the Problem 2 data set to allow participants to address complementary questions in a data set with a known genetic architecture. Here we provide a brief summary of the data sets; further details can be found in Cheung and Spielman [1], Amos et al. [2], and Miller et al. [3] in this issue.

The Problem 1 data set included microarray expression profiles originally investigated by Morley at el. [4]. Data were provided for 14 three-generation Centre d'Etude du Polymorphisme Humain (CEPH) Utah families (approximately 8 offspring per sibship and approximately 14 individuals per family). Phenotypes included expression level of genes in lymphoblastoid cells of these family members, obtained using the Affymetrix Human Focus Arrays that contain probes for 8500 transcripts. Among these, Morley et al. [4] found greater variation among individuals than between replicate determinations on the same individual for 3554 expression phenotypes (expressed genes); these were provided to GAW15. For approximately 100 individuals, array hybridizations were performed in duplicate. The Affymetrix CEL files for all array hybridizations were provided to GAW15 participants. Genotypes for members of the 14 families were provided for 2882 autosomal and X-linked single-nucleotide polymorphisms (SNPs). The genotypes were generated by The SNP Consortium. This data set provided the opportunity to develop and apply methods for simultaneous analysis of a variety of related traits. Natural variation in gene expression is a new idea, and this collection is the first to provide such a large number of phenotypes in a family study.

The Problem 2 data set consisted of family- and population-based data from the North American Rheumatoid Arthritis Consortium study (NARAC) and from collaborators in Canada, France, and England. The goal of these studies is to understand the etiology of rheumatoid arthritis. It is highly likely that multiple interacting loci influence disease risk, as evidenced by the considerably higher recurrence risk for this disease to siblings as opposed to more distant relatives. The data provided by NARAC to GAW15 included 757 multiplex families genome scanned with microsatellites (511 families) and/or SNPs (746 families), candidate gene data for the *PTPN22 *locus from a study of 1519 controls and 1393 cases (and for additional candidate loci in a separate sample of 855 controls and 839 cases), dense genotyping data from a panel of 2300 SNPs for an approximately 10-kb region of chromosome 18q (genotyped on 460 cases and 460 controls), and further data on a number of quantitative phenotypes and clinical measures. The Canadian group provided data from 60 families that had been genotyped using the same Illumina platform used by NARAC as well as 79 families that were genotyped using an Affymetrix 100 K platform. The European Consortium on Rheumatoid Arthritis Families provided high-density microsatellite data from 88 families typed with 1089 microsatellite markers. The UK group provided microsatellite genome screen data from 174 families, of which 157 were also genotyped at 10,156 SNPs. A further set of 195 families genotyped at selected microsatellites was also provided.

The Problem 3 data set included 100 replicates of simulated data, modeled after the rheumatoid arthritis data set. Each replicate included 1500 nuclear families of size four (two parents and an affected sib pair (ASP)) and 2000 unrelated controls. Three sets of autosomal markers were generated: 1) a set of 730 microsatellite markers spaced on average 5 cM apart; 2) a set of 9187 SNPs distributed on the genome to mimic a 10 K SNP chip set; and 3) a very dense map of 17820 SNPs on chromosome 6 (an average inter-marker spacing of 9586 bp). The data included map information, with lists of markers and their locations, and simulated family, marker, and phenotype/covariate data. "Answers" (the locations/effects of true causal loci and a description of the underlying generating model) were provided to GAW15 participants on request.

The availability of the GAW15 data was announced by email in the Spring of 2006, to the more than 2600 individuals on the GAW mailing list. A total of 179 groups requested GAW15 data. The Problem 1 data were requested by 133 groups, the Problem 2 data by 142 groups, and the Problem 3 data by 128 groups (with many groups requesting access to more than one data set). In the Summer of 2006, 252 contributed papers were received describing analyses of these data sets. A book and CD containing these contributions plus descriptions of the data sets were distributed to GAW15 participants.

The GAW15 participants included 350 individuals from 20 countries on four continents – Asia, Australia, Europe, North and South America. The 252 contributions submitted to GAW15 were organized into 17 presentation groups of 11 to 18 papers each, grouped based on common methodological themes. The 17 presentation groups were organized around the following themes: association analysis (Problem 1); association analysis (Problem 2); association analysis (Problem 3); combining linkage and association; data mining, neural networks, and gene networks (Problem 1); data mining, neural networks, and trees (Problems 2 and 3); gene × gene interaction; gene × environment interaction; linkage analysis (Problem 1); linkage analysis (Problems 2 and 3); model selection and Bayesian methods; multivariate analysis; candidate gene association; multistage designs; multiple testing and false discovery rate; processing and normalization of expression traits and their effect on analysis; and SNP selection, ancestry informative markers, and linkage disequilibrium between markers. For each presentation group, a group leader was chosen who had previous GAW experience. This person facilitated group discussion, organized the group's oral presentation to the general GAW meeting, and took the lead in writing the group summary paper to be published in *Genetic Epidemiology*.

Members of most presentation groups began interacting by email and/or conference call before GAW15, comparing and contrasting their approaches and results. Each presentation group also met at least once during the Workshop, where they continued their discussions and finalized a group presentation that was delivered to the full GAW15 audience during the general sessions. The group meetings were attended mostly by group participants but were open to all GAW15 attendees. During poster sessions, 118 individual contributions were presented. There also was a special general session on "Novel Methods" at which four of the contributions (selected prior to GAW15 on the basis of the submitted papers) that had used or developed novel analytical approaches were highlighted and presented.

The 162 GAW contributions included in this issue of *BMC Proceedings *are a subset of the 252 contributions presented at GAW15. All of these papers have been peer-reviewed and were selected on the basis of scientific merit. First come three papers that describe the data sets. These are followed by the 162 individual GAW15 contributions organized by presentation group, and alphabetically by first author within each group. Additionally, in a forthcoming supplement to the journal *Genetic Epidemiology*, a paper by each presentation group summarizes the contributions to that group and the lessons learned, comparing and contrasting contributions and describing their main themes and results. Overall, GAW15 generated many interesting discussions and some conclusions concerning appropriate approaches to the analysis of massively multivariate data, and methods for separating multiple functional loci within a region of linkage or association. These discussions also highlighted areas in which further methodological development is needed.

## Competing interests

The author(s) declare that they have no competing interests.
